# The Challenge of Diagnosing and Managing Pulmonary Arterial Hypertension in Systemic Sclerosis with Interstitial Lung Disease

**DOI:** 10.3390/ph15091042

**Published:** 2022-08-24

**Authors:** Elisabetta Zanatta, Martina Perazzolo Marra, Giulia Famoso, Elisabetta Balestro, Chiara Giraudo, Fiorella Calabrese, Federico Rea, Andrea Doria

**Affiliations:** 1 Department of Medicine-DIMED, Padova University Hospital, Via Giustiniani 2, 35126 Padova, Italy; 2 Department of Cardiac, Thoracic, Vascular Sciences and Public Health, Padova University Hospital, Via Giustiniani 2, 35126 Padova, Italy

**Keywords:** systemic sclerosis, pulmonary arterial hypertension, interstitial lung disease

## Abstract

Pulmonary hypertension (PH) in patients with Systemic Sclerosis (SSc) may stem from a variety of underlying causes, thus making a correct diagnosis and management difficult. The main challenges lie in the distinction between pulmonary arterial hypertension (PAH, group 1) and PH due to interstitial lung disease (PH-ILD, group 3) in patients with concomitant lung fibrosis — a very common occurrence in SSc. A consensus among experts remains elusive. Some studies have suggested that among SSc patients with PH, those with an ILD extension > 20% at high-resolution computed tomography (HRCT) should be considered as affected by PH-ILD, whereas other Authors have found that a wide proportion of these patients exhibit features of both PAH and group 3 PH-ILD. We report the case of a 46-year-old male SSc patient with a stable and extensive ILD (>20%) who developed a histologically documented pulmonary vasculopathy typical of PAH and received PAH-specific treatment as bridge to transplant. Moreover, we documented PH disease course by right heart catheterization (RHC), with and without specific vasodilator therapies, which are essential in PAH but not indicated and/or harmful in PH-ILD.

## 1. Introduction

Pulmonary hypertension (PH) is a haemodynamic state caused by several pathophysiological conditions. Patients affected by systemic sclerosis (SSc) may present a wide variability of causes for PH, sometimes concomitantly, thus making a correct diagnosis and management difficult [1,2]. Whereas a timely and aggressive vasodilatory treatment is essential in pulmonary arterial hypertension (PAH, group 1), it is not indicated and/or may be harmful in PH due to left heart disease (group 2), interstitial lung disease (PH-ILD, group 3), and pulmonary veno-occlusive disease (group 4) [3]. The main challenge is the distinction between PAH and PH-ILD in patients with concomitant lung fibrosis—a very common occurrence in SSc. A consensus among experts remains elusive, as they are both precapillary conditions AT right heartcatheterisation (RHC). Some authors and a cluster analysis performed in precapillary PH-SSc suggested that patients with defined PH at RHC and ILD extension > 20%, assessed via high-resolution computed tomography (HRCT), should be considered as affected by group 3 PH-ILD [4], thus avoiding vasodilator therapies. However, a recent American cohort study [5] reported that a wide proportion of patients with >20% extent of ILD on HRCT showed feature of both PAH and group 3 PH-ILD; hence, individuals within this group were treated with both vasodilators and immunosuppressants on a case-by-case basis, resulting in a remarkably high survival rate (91% at 3 years).

## 2. Case Presentation

**Figure 1:**
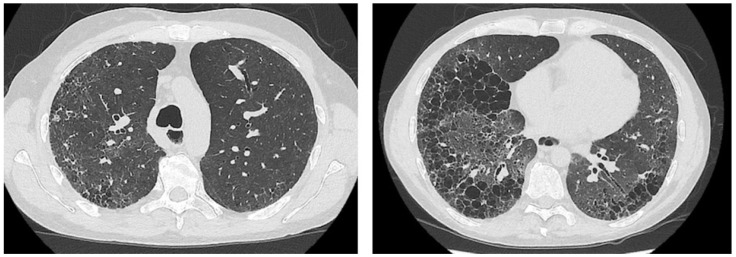
Axial High-Resolution Computed Tomography (HRCT) images showing a typical Usual Interstitial Pneumoniae (UIP) pattern that consists of predominantly basal and peripheral reticular opacities and honeycombing with traction bronchiectasis.

We report the case of a 46-year-old man affected with SSc since 2011, with anti-RNA-polymerase-III positivity, diffuse cutaneous involvement and extensive/progressive ILD. He was treated with mycophenolate mofetil (discontinued after few months due to severe lung infection), low-dose prednisone, and rituximab (1 g × 2 infusions every 6–8 months) with a significant improvement of the modified Rodnan skin score (from 21 to 9) and ILD stabilisation in early 2014. The radiological aspect was defined as the usual interstitial pneumonia (UIP) pattern, involving about 30% of the lung parenchyma (i.e., extensive ILD, Figure 1). Nevertheless, he presented only a moderate dyspnoea (NYHA II), without any signs of PH on transthoracic Doppler echocardiography (TTE).

**Figure 2:**
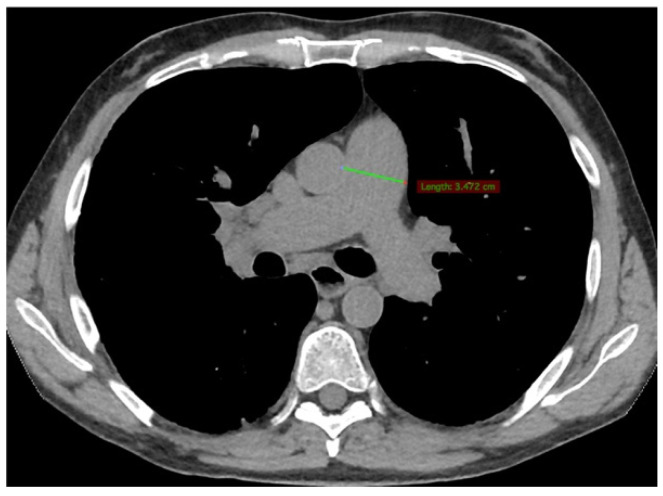
Dilated pulmonary artery trunk at repeated HRCT.

In May 2015, the patient presented with worsening dyspnoea (NYHA III); pulmonary function tests (PFTs) showed stable restrictive lung disease (forced vital capacity: 74% predicted; total lung capacity: 71% predicted) with a reduction in diffusing capacity for carbon monoxide (DLCO: 56% to 34% predicted). Repeated HRCT showed stable ILD, with dilated pulmonary artery trunk (diameter: 3.47 centimetres; Figure 2); TTE revealed a systolic pulmonary artery pressure (PAP) of 58 mmHg, a moderately dilated right ventricle (end diastolic volume, EDV 90 mL/m^2^) with reduced systolic function (tricuspid annular plane systolic excursion, TAPSE 1.21 cm); left ventricle volume and ejection fraction (69%) were normal.

The RHC was performed with evidence of precapillary PH (mean PAP: 42 mmHG; wedge pressure: 7 mmHg; arteriolar resistance: 6.7 WU) and cardiac index (CI) within normal range; a pulmonary angiography ruled out embolism. The diagnosis of PAH with concomitant ILD was established following a multidisciplinary discussion, which highlighted the new onset of PH in the presence of stable lung involvement. Treatment was promptly initiated with endothelin receptor antagonist (ERA), resulting in clinical and echocardiographic improvements.

**Figure 3:**
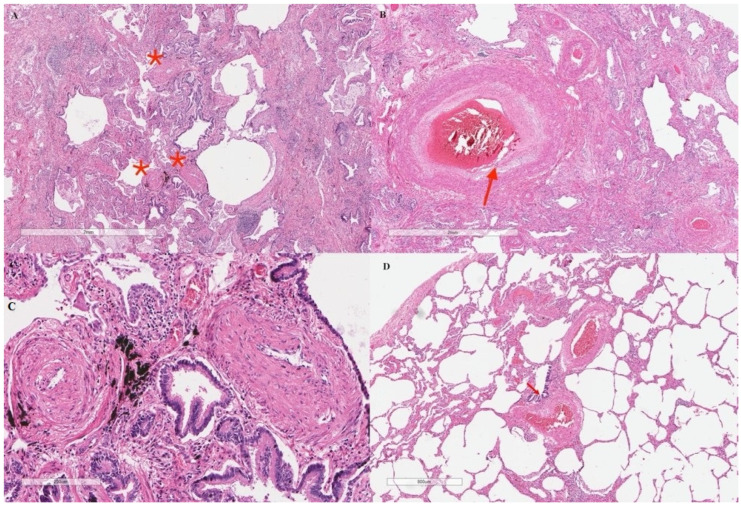
Histologic examination of the explanted lungs. Typical usual interstitial pneumonia pattern (UIP) underlying autoimmune disease with peribronchial metaplasia, interstitial fibrosis, nodular lymphocytic infiltration, and micro-honeycombing. Vascular remodelling throughout the lung parenchyma (stars on vessels) (**A**), haematoxylin and eosin stain (×20). Large/mid-sized arteries with diffuse aspects of medio-intimal hyperplasia (**B**), haematoxylin and eosin stain (×20). Small vessels showing medial hypertrophy and intimal fibrosis, leading to lumen narrowing (**C**), haematoxylin and eosin stain (×100). Vascular changes (arrow) were found in more preserved areas as well (**D**), haematoxylin and eosin stain (×20).

Six months later, the patient’s dyspnoea had progressed without signs of heart failure and/or ILD progression. Surprisingly, a repeat RHC showed a severely increased wedge pressure of 34 mmHg. Suspicion of undiagnosed occult left heart disease (LHD)—a quite common occurrence in SSc [6,7]—prompted ERA discontinuation. A cardiac resonance imaging ruled out LHD and, upon the further deterioration of the patient’s condition, a repeated RHC revealed a normal end-diastolic pressure and wedge pressure (6 mmHg and 8 mmHg, respectively), thus suggesting a previous wedging error, as it has been reported [8]. Notably, arterial resistance was remarkably increased (21.5 WU) with a low CI (1.83 l/min/mq). Treatment with ERA was reinitiated, and a phosphodiesterase inhibitor was added with clinical and RHC improvement (arteriolar resistance: 9 WU (with normal CI)), and without a worsening of hypoxaemia. Given the severity of ILD and PAH, and the absence of specific SSc-related contraindications [9], the patient underwent bilateral lung transplantation in August 2019. The histological examination of explanted lungs confirmed a concomitant UIP pattern and typical PAH vasculopathy (Figure 3), which is histologically different from vascular remodelling due to hypoxaemia (PH-ILD).

## 3. Discussion and Conclusions

Interstitial lung disease is a very common occurrence in SSc, detectable in up to 70% of patients. Therefore, unlike in the idiopathic form of PAH, concomitant group 1 PAH-SSc and ILD are not rare and should be considered. Although there is no consensus on how to distinguish PAH from group 3 PH-LD, patients with ILD extension > 20% of the lung parenchyma and PH are generally classified as group 3, and thus vasodilatory treatments are avoided [4]. Nevertheless, a large American cohort study [5] recently found that some SSc patients with extensive ILD exhibited features reminiscent of PAH and patients were treated with vasodilators and/or immunosuppressants, resulting in a remarkably high 3-year survival. It should be noted that this study did not provide clear evidence of PAH vasculopathy.

As far as we know, ours is the first histological demonstration of a real pulmonary vasculopathy typical of PAH in an SSc patient with extensive ILD. This corroborated reports by Young A et al. [5] by providing robust evidence that a real PAH may sometimes coexist with extensive ILD in SSc. Therefore, this possibility must be considered, especially when pulmonary fibrosis is stable, as previously suggested by Fayed et al. [10]. In our case, the main red flags for PAH were the development within few months of clear indirect signs of PH on TTE with a drop in DLCO on PFTs, despite lung volumes comparable to the previous ones and complete radiological stability of ILD on repeat HRCT. Moreover, RHC confirmed severe mPAP with reduced CI. These findings prompted us to initiate PAH-specific therapy as a bridge to lung transplantation. Due to a wedging error [8], we also had the unique opportunity to document the PH disease course, through RHC, with and without specific vasodilator therapies. The dramatic increase in arteriolar resistance translated into rapid clinical worsening following ERA discontinuation, suggesting that our patient may not have survived until lung transplantation without PAH-specific therapy.

In conclusion, our case demonstrated that PAH vasculopathy may sometimes coexist with extensive ILD in SSc patients. This possibility must be considered in the presence of new onset PH notwithstanding a completely stable pulmonary fibrosis. Considering the high frequency of ILD in SSc and the poor prognosis in patients with PAH, a multidisciplinary approach in specialised centres is paramount to identify patients with PAH and ILD who may benefit most from specific vasodilatory therapies. In these challenging SSc cases, patient-tailored treatments should be considered for a better prognosis [5,11].

## Data Availability

Data is contained within the article.
